# Poisson-type inequalities for growth properties of positive superharmonic functions

**DOI:** 10.1186/s13660-016-1278-7

**Published:** 2017-01-06

**Authors:** Kuan Luan, John Vieira

**Affiliations:** 1 Automation School, Harbin Engineering University, Harbin, 150001 China; 2 Department of Matemática, Universidade Federal de Minas Gerais, Av Antonio Carlos 6627, Caixa Postal 702, Belo Horizonte, MG BR-31270901 Brazil

**Keywords:** Poisson-type inequality, continuous data, growth property

## Abstract

In this paper, we present new Poisson-type inequalities for Poisson integrals with continuous data on the boundary. The obtained inequalities are used to obtain growth properties at infinity of positive superharmonic functions in a smooth cone.

## Introduction

Cartesian coordinates of a point *G* of $\mathbf{R}^{n}$, $n\geq2$, are denoted by $(X,x_{n})$, where $\mathbf{R}^{n}$ is the *n*-dimensional Euclidean space and $X=(x_{1},x_{2},\ldots,x_{n-1})$. We introduce spherical coordinates for $G=(r,\Xi)$ ($\Xi=(\theta_{1},\theta _{2},\ldots ,\theta_{n-1})$) by $|x|=r$,

$$\left \{ \textstyle\begin{array}{l@{\quad}l} x_{n}=r\cos\theta_{1}, \qquad x_{1}=r(\prod_{j=1}^{n-1}\sin\theta_{j}),& n= 2, \\ x_{n-m+1}=r(\prod_{j=1}^{m-1}\sin\theta_{j})\cos\theta_{m}, & n\geq3, \end{array}\displaystyle \right . $$

where $0\leq r<+\infty$, $-\frac{1}{2}\pi\leq\theta_{n-1}<\frac{3}{2}\pi$ and $0\leq\theta_{j}\leq\pi$ for $1\leq j\leq n-2$ ($n\geq3$).

We denote the unit sphere and the upper half unit sphere by $\mathbf{ S}^{n-1}$ and $\mathbf{S}_{+}^{n-1}$, respectively. Let $\Sigma\subset \mathbf{ S}^{n-1}$. The point $(1,\Xi)$ and the set $\{\Xi; (1,\Xi)\in\Sigma\}$ are identified with Ξ and Σ, respectively. Let $\Xi\times\Sigma$ denote the set $\{(r,\Xi)\in\mathbf{R}^{n}; r\in\Xi,(1,\Xi)\in\Sigma\}$, where $\Xi\subset\mathbf{R}_{+}$. The set $\mathbf{R}_{+}\times\Sigma$ is denoted by $\beth_{n}(\Sigma)$, which is called a cone. Especially, the set $\mathbf{ R}_{+}\times\mathbf{S}_{+}^{n-1}$ is called the upper-half space, which is denoted by $\mathcal{T}_{n}$. Let $I\subset\mathbf{R}$. Two sets $I\times\Sigma$ and $I\times\partial{\Sigma}$ are denoted by $\beth_{n}(\Sigma;I)$ and $\daleth_{n}(\Sigma;I)$, respectively. We denote $\daleth_{n}(\Sigma; \mathbf{R}^{+})$ by $\daleth_{n}(\Sigma)$, which is $\partial{\beth_{n}(\Sigma)}-\{O\}$.

Let $B(G,l)$ denote the open ball, where $G\in\mathbf{R}^{n}$ is the center and $l>0$ is the radius.

### Definition 1

Let *E* be a subset of $\beth _{n}(\Sigma )$. If there exists a sequence of balls $\{B_{k}\}$ ($k=1,2,3,\ldots$) with centers in $\beth_{n}(\Sigma)$ satisfying

$$E\subset\bigcup_{k=0}^{\infty} B_{k}, $$

then we say that *E* has a covering $\{r_{k},R_{k}\}$, where $r_{k}$ is the radius of $B_{k}$ and $R_{k}$ is the distance from the origin to the center of $B_{k}$ (see [1]).

In spherical coordinate the Laplace operator is

$$\Delta_{n}=r^{-2}\Lambda_{n}+r^{-1}(n-1) \frac{\partial}{\partial r}+\frac{\partial^{2}}{\partial r^{2}}, $$

where $\Lambda_{n}$ is the Beltrami operator. Now we consider the boundary value problem

$$\begin{aligned} &(\Lambda_{n}+\tau)h=0 \quad\text{on } \Sigma,\\ &h=0\quad \text{on } \partial{\Sigma}. \end{aligned} $$

If the least positive eigenvalue of it is denoted by $\tau_{\Sigma}$, then we can denote by $h_{\Sigma}(\Xi)$ the normalized positive eigenfunction corresponding to it.

We denote by $\iota_{\Sigma}$ (>0) and $-\kappa_{\Sigma}$ (<0) two solutions of the problem $t^{2}+(n-2)t-\tau_{\Sigma}=0$, Then $\iota _{\Sigma}+\kappa_{\Sigma}$ is denoted by $\varrho_{\Sigma}$ for the sake of simplicity.

### Remark 1

In the case $\Sigma=\mathbf{S}_{+}^{n-1}$, it follows that

(I) $\iota_{\Sigma}=1$ and $\kappa_{\Sigma}=n-1$.
(II) $h_{\Sigma}(\Xi)=\sqrt{\frac{2n}{ w_{n}}}\cos\theta_{1}$, where $w_{n}$ is the surface area of $\mathbf{S}^{n-1}$.

It is easy to see that the set $\partial{\beth_{n}(\Sigma)}\cup\{\infty\}$ is the Martin boundary of $\beth_{n}(\Sigma)$. For any $G\in\beth_{n}(\Sigma)$ and any $H\in\partial{\beth_{n}(\Sigma)}\cup\{\infty\}$, if the Martin kernel is denoted by $\mathcal{MK}(G,H)$, where a reference point is chosen in advance, then we see that (see [2])

$$\mathcal{MK}(G,\infty)=r^{\iota_{\Sigma}}h_{\Sigma}(\Xi )\quad \text{and}\quad \mathcal{MK}(G,O)=cr^{-\kappa_{\Sigma}}h_{\Sigma}(\Xi), $$

where $G=(r,\Xi)\in \beth_{n}(\Sigma)$ and *c* is a positive real number.

We shall say that two positive real valued functions *f* and *g* are comparable and write $f\approx g$ if there exist two positive constants $c_{1}\leq c_{2}$ such that $c_{1}g\leq f\leq c_{2}g$.

### Remark 2

Let $\Xi\in\Sigma$. Then $h_{\Sigma}(\Xi)$ and $\operatorname{dist}(\Xi,\partial{\Sigma})$ are comparable.

### Remark 3

Let $\varrho(G)=\operatorname{dist}(G,\partial{\beth_{n}(\Sigma)})$. Then $h_{\Sigma}(\Xi)$ and $\varrho(G) $ are comparable for any $(1,\Xi)\in\Sigma$ (see [3]).

### Remark 4

Let $0\leq\alpha\leq n$. Then $h_{\Sigma}(\Xi)\leq c_{3}(\Sigma,n)\{h_{\Sigma}(\Xi)\}^{1-\alpha}$, where $c_{3}(\Sigma,n)$ is a constant depending on Σ and *n* (*e.g.* see [4], pp.126-128).

### Definition 2

For any $G\in\beth_{n}(\Sigma)$ and any $H\in\beth_{n}(\Sigma)$. If the Green function in $\beth_{n}(\Sigma)$ is defined by $\mathcal {GF}_{\Sigma }(G,H)$, then:

(I) The Poisson kernel can be defined by
  $$\mathcal{POI}_{\Sigma}(G,H)=\frac{\partial}{\partial n_{H}}\mathcal{GF}_{\Sigma}(G,H), $$
  where $\frac{\partial}{\partial n_{H}}$ denotes the differentiation at *H* along the inward normal into $\beth_{n}(\Sigma)$.
(II) The Green potential in $\beth_{n}(\Sigma)$ can be defined by
  $$\mathcal{GF}_{\Sigma} \nu(G)= \int_{\beth_{n}(\Sigma)}\mathcal{GF}_{\Sigma}(G,H)\,d\nu(H), $$
  where $G\in \beth_{n}(\Sigma)$ and *ν* is a positive measure in $\beth_{n}(\Sigma)$.

### Definition 3

For any $G\in\beth_{n}(\Sigma)$ and any $H\in\daleth_{n}(\Sigma)$. Let *μ* be a positive measure on $\daleth_{n}(\Sigma)$ and *g* be a continuous function on $\daleth_{n}(\Sigma)$. Then:

(I) The Poisson integral with *μ* can be defined by
  $$\mathcal{POI}_{\Sigma} \mu(G)= \int_{\daleth_{n}(\Sigma)}\mathcal{POI}_{\Sigma}(G,H)\,d\mu(H). $$
(II) The Poisson integral with *g* can be defined by
  $$\mathcal{POI}_{\Sigma} [g](G)= \int_{\daleth_{n}(\Sigma)}\mathcal{POI}_{\Sigma }(G,H)g(H)\,d \sigma_{H}, $$
  where $d\sigma_{H}$ is the surface area element on $\daleth_{n}(\Sigma)$.

### Definition 4

Let *μ* be defined in Definition 3. Then the positive measure $\mu'$ is defined by

$$d\mu'=\left \{ \textstyle\begin{array}{l@{\quad}l} \frac{\partial h_{\Sigma}(\Omega)}{\partial n_{\Omega}} t^{-\kappa _{\Sigma}-1}\,d\mu& \mbox{on } \daleth_{n}(\Sigma; (1,+\infty)) ,\\ 0 & \mbox{on } \mathbf{R}^{n}-\daleth_{n}(\Sigma; (1,+\infty)). \end{array}\displaystyle \right . $$

### Definition 5

Let *ν* be any positive measure in $\beth_{n}(\Sigma)$ satisfying

1 $$ \mathcal{GF}_{\Sigma} \nu(G)\not\equiv+\infty $$

for any $G\in\beth_{n}(\Sigma)$. Then the positive measure $\nu'$ is defined by

$$d\nu'=\left \{ \textstyle\begin{array}{l@{\quad}l} h_{\Sigma}(\Omega) t^{-\kappa_{\Sigma}} \,d\nu& \mbox{on } \beth _{n}(\Sigma; (1,+\infty)) ,\\ 0& \mbox{on } \mathbf{R}^{n}-\beth_{n}(\Sigma; (1,+\infty)). \end{array}\displaystyle \right . $$

### Definition 6

Let *μ* and *ν* be defined in Definitions 3 and 4, respectively. Then the positive measure *ξ* is defined by

$$d\xi=\left \{ \textstyle\begin{array}{l@{\quad}l} t^{-1-\kappa_{\Sigma}}\, d\xi' & \mbox{on } \overline{\beth _{n}(\Sigma ; (1,+\infty))} ,\\ 0& \mbox{on } \mathbf{R}^{n}-\overline{\beth_{n}(\Sigma; (1,+\infty))}, \end{array}\displaystyle \right . $$

where

$$d\xi'=\left \{ \textstyle\begin{array}{l@{\quad}l} \frac{\partial h_{\Sigma}(\Omega)}{\partial n_{\Omega}}\,d\mu(H) & \mbox{on } \daleth_{n}(\Sigma; (1,+\infty)) ,\\ h_{\Sigma}(\Omega)t\,d\nu(H)& \mbox{on } \beth_{n}(\Sigma; (1,+\infty)). \end{array}\displaystyle \right . $$

### Remark 5

Let $\Sigma=\mathbf{S}_{+}^{n-1}$. Then

$$\mathcal{GF}_{\mathbf{S}_{+}^{n-1}}(G,H)=\left \{ \textstyle\begin{array}{l@{\quad}l} \log|G-H^{\ast}|-\log|G-H| & \mbox{if } n=2, \\ |G-H|^{2-n}-|G-H^{\ast}|^{2-n} & \mbox{if } n\geq3, \end{array}\displaystyle \right . $$

where $G=(X,x_{n})$, $H^{\ast}=(Y,-y_{n})$, that is, $H^{\ast}$ is the mirror image of $H=(Y,y_{n})$ on $\partial{\mathcal{T}_{n}}$. Hence, for the two points $G=(X,x_{n})\in\mathcal{T}_{n}$ and $H=(Y,y_{n})\in\partial {\mathcal {T}_{n}}$, we have

$$\mathcal{POI}_{\mathbf{S}_{+}^{n-1}}(G,H)=\frac{\partial}{\partial n_{y}}\mathcal{GF}_{\mathbf{S}_{+}^{n-1}}(G,H)= \left \{ \textstyle\begin{array}{l@{\quad}l} 2x_{n}|G-H|^{-2} & \mbox{if } n=2, \\ 2(n-2)x_{n}|G-H|^{-n} & \mbox{if } n\geq3. \end{array}\displaystyle \right . $$

### Remark 6

Let $g(H)$ be a continuous function on $\daleth_{n}(\Sigma)$. If $d\mu =|g|\,d\sigma_{H}$, then we define

$$d\mu''=\left \{ \textstyle\begin{array}{l@{\quad}l} \frac{\partial h_{\Sigma}(\Omega)}{\partial n_{\Omega }}|g|t^{-1-\kappa _{\Sigma}}\,d\sigma_{H} & \mbox{on } \daleth_{n}(\Sigma; (1,+\infty)) ,\\ 0& \mbox{on } \mathbf{R}^{n}-\daleth_{n}(\Sigma; (1,+\infty)). \end{array}\displaystyle \right . $$

### Remark 7

Let $\Sigma=\mathbf{S}_{+}^{n-1}$. Then we define

$$d\varrho=\left \{ \textstyle\begin{array}{l@{\quad}l} \frac{d\varrho'}{|y|^{n}} & \mbox{on } \overline{\mathcal {T}_{n}} ,\\ 0& \mbox{on } \mathbf{R}^{n}-\overline{\mathcal{T}_{n}}, \end{array}\displaystyle \right . $$

where

$$d\varrho'(y)=\left \{ \textstyle\begin{array}{l@{\quad}l} d\mu& \mbox{on } \partial{\mathcal{T}_{n}} ,\\ y_{n}d\nu& \mbox{on } \mathcal{T}_{n}. \end{array}\displaystyle \right . $$

### Definition 7

Let *λ* be any positive measure on $\mathbf{R}^{n}$ having finite total mass. Then the maximal function $M(G;\lambda,\beta)$ is defined by

$$\mathfrak{M}(G;\lambda,\beta)=\sup_{ 0< \rho< \frac{r}{2}}\rho^{-\beta} \lambda\bigl(B(G,\rho)\bigr) $$

for any $G=(r,\Xi)\in \mathbf{R}^{n}-\{O\}$, where $\beta\geq0$. The exceptional set can be defined by

$$\mathbb{EX}(\epsilon; \lambda, \beta)=\bigl\{ G=(r,\Xi)\in\mathbf{R}^{n}- \{O\}; \mathfrak{M}(G;\lambda,\beta)r^{\beta}>\epsilon\bigr\} , $$

where *ϵ* is a sufficiently small positive number.

### Remark 8

Let $\beta>0$ and $\lambda(\{P\})>0$ for any $P\neq O$. Then

(I) Then $\mathfrak{M}(G;\lambda,\beta)=+\infty$.
(II) $\{G\in\mathbf{R}^{n}-\{O\}; \lambda(\{P\})>0\}\subset \mathbb{EX}(\epsilon; \lambda, \beta)$.

Recently, Qiao and Wang (see [5], Corollary 2.1 with $m=0$) proved classical Poisson-type inequalities for Poisson integrals in a half space. Applications of them were also developed by Pang and Ychussie (see [6]) and Xue and Wang (see [7]). In particular, Huang (see [8]) further obtained Schrödinger-Poisson-type inequalities for Poisson-Schrödinger integrals and gave their related applications.

### Theorem A

*Let* *g* *be a measurable function on* $\partial{\mathcal{T}_{n}}$ *satisfying*

2 $$ \int_{\partial{\mathcal{T}_{n}}}\bigl|g(y)\bigr|\bigl(1+|y|\bigr)^{-n} \,dy< \infty. $$

*Then the harmonic function* $\mathcal{POI}_{\mathbf{S}_{+}^{n-1}}[g](x)=\int_{\partial{\mathcal {T}_{n}}}\mathcal{POI}_{\mathbf{S}_{+}^{n-1}}(x,y)g(y)\,dy$ *satisfies*

3 $$ \mathcal{POI}_{\mathbf{S}_{+}^{n-1}}[g]=o\bigl(|x|\sec^{n-1} \theta_{1}\bigr) $$

*as* $|x|\rightarrow\infty$ *in* $\mathcal{T}_{n}$.

## Results

Our first aim in this paper is to prove the following result, which is a generalization of Theorem A. For similar results with respect to Schrödinger operator, we refer the reader to the literature (see [5, 9]).

### Theorem 1

*Let* $\mathcal{POI}_{\Sigma}\mu (G)\not \equiv +\infty$ *for any* $G=(r,\Xi)\in\beth_{n}(\Sigma)$, *where* *μ* *is a positive measure on* $\daleth_{n}(\Sigma)$. *Then*

4 $$ \mathcal{POI}_{\Sigma} \mu(G)=o\bigl(r^{\iota_{\Sigma}}\bigl\{ h_{\Sigma}(\Xi)\bigr\} ^{1-\alpha}\bigr), $$

*for any* $G\in\beth_{n}(\Sigma)-\mathbb{EX}(\epsilon; \mu',n-\alpha)$ *as* $r \rightarrow\infty$, *where* $\mathbb {EX}(\epsilon; \mu',n-\alpha)$ *is a subset of* $\beth_{n}(\Sigma)$ *and has a covering* $\{r_{k},R_{k}\}$ *of satisfying*

5 $$ \sum_{k=0}^{\infty}\biggl( \frac{r_{k}}{R_{k}}\biggr)^{n-\alpha}< \infty. $$

Let $d\mu=|g|\,d\sigma_{H}$ for any $H=(t,\Omega)\in\daleth_{n}(\Sigma )$. Then we have the following result, which generalizes Theorem A to the conical case.

### Corollary 1

*If* *g* *is a measurable function on* $\daleth_{n}(\Sigma)$ *satisfying*

6 $$ \int_{1}^{\infty}\frac{\int_{\partial{\Sigma}}|g(H)|\,d_{\sigma _{\Omega}}}{t^{1+\iota_{\Sigma}}}\,dt< \infty. $$

*Then the Poisson integral* $\mathcal{POI}_{\Sigma}[g](G)$ *is harmonic in* $\beth_{n}(\Sigma)$ *and*

7 $$ \mathcal{POI}_{\Sigma}[g](G)=o\bigl(r^{\iota_{\Sigma}}\bigl\{ h_{\Sigma}(\Xi)\bigr\} ^{1-\alpha}\bigr) $$

*for any* $G\in\beth_{n}(\Sigma)- \mathbb{EX}(\epsilon; \mu'',n-\alpha)$ *as* $r \rightarrow\infty$, *where* $\mathbb {EX}(\epsilon ; \mu'',n-\alpha)$ *is a subset of* $\beth_{n}(\Sigma)$ *and has a covering* $\{r_{k},R_{k}\}$ *satisfying* (5).

### Remark 9

If $\Sigma=\mathbf{S}_{+}^{n-1}$, then it is easy to see that (6) is equivalent to (2) and (5) is a finite sum, then the set $\mathbb{EX}(\epsilon; \mu'',0)$ is a bounded set and (7) reduces to (3) in the case $\alpha=n$ from Remark 1.

Let $\Sigma=\mathbf{S}_{+}^{n-1}$. We immediately have the following results from Theorem 1.

### Corollary 2

*If* *μ* *is a positive measure on* $\partial{\mathcal{T}_{n}}$ *satisfying* $\mathcal{POI}_{\mathbf{S}_{+}^{n-1}}\mu(x)\not\equiv+\infty$ *for any* $x=(X,x_{n})\in\mathcal{T}_{n}$, *then*

$$\mathcal{POI}_{\mathbf{S}_{+}^{n-1}} \mu(x)=0\bigl(|x|\bigr) $$

*for any* $x\in \mathcal{T}_{n}-\mathbb{EX}(\epsilon;\mu',n-1)$ *as* $|x| \rightarrow \infty $, *where* $\mathbb{EX}(\epsilon;\mu',n-1)$ *is a subset of* $\beth _{n}(\Sigma)$ *and has a covering* $\{r_{k},R_{k}\}$ *satisfying*

8 $$ \sum_{k=0}^{\infty}\biggl( \frac{r_{k}}{R_{k}}\biggr)^{n-1}< \infty. $$

### Corollary 3

*Let* *μ* *be defined as in Corollary* 2. *Then*

$$\mathcal{POI}_{\mathbf{S}_{+}^{n-1}} \mu(x)=0(x_{n}) $$

*for any* $x\in \mathcal{T}_{n}-\mathbb{EX}(\epsilon;\mu',n)$ *as* $|x| \rightarrow \infty $, *where* $\mathbb{EX}(\epsilon;\mu',n)$ *is a subset of* $\beth _{n}(\Sigma )$ *and has a covering* $\{r_{k},R_{k}\}$ *satisfying*

9 $$ \sum_{k=0}^{\infty}\biggl( \frac{r_{k}}{R_{k}}\biggr)^{n}< \infty. $$

The following result is very well known. We quote it from [10].

### Theorem B

see [10]

*Let* $0< w(G)$ *be a superharmonic function in* $\mathcal{T}_{n}$. *Then there exist a positive measure* *μ* *on* $\partial\mathcal{T}_{n}$ *and a positive measure* *ν* *on* $\mathcal{T}_{n}$ *such that* $w(x)$ *can be uniquely decomposed as*

10 $$ w(x)=cx_{n}+\mathcal{POI}_{\mathbf{S}_{+}^{n-1}} \mu(x)+ \mathcal{GF}_{\mathbf{S}_{+}^{n-1}} \nu(x), $$

*where* $x=(X,X_{n})\in\mathcal{T}_{n}$ *and* *c* *is a nonnegative constant*.

### Theorem C

see [9], Theorem 2

*Let* $0< w(G)$ *be a superharmonic function in* $\beth_{n}(\Sigma)$. *Then there exist a positive measure* *μ* *on* $\daleth_{n}(\Sigma)$ *and a positive measure* *ν* *in* $\beth_{n}(\Sigma)$ *such that* $w(G)$ *can be uniquely decomposed as*

11 $$ w(G)=c_{5}(w)\mathcal{MK}(G,\infty)+c_{6}(w) \mathcal{MK}(G,O)+\mathcal {POI}_{\Sigma} \mu(G)+\mathcal{GF}_{\Sigma} \nu(G), $$

*where* $G\in\beth_{n}(\Sigma)$, $c_{5}(w)$, *and* $c_{6}(w)$ *are two constants dependent of* *w* *satisfying*

$$c_{5}(w)=\inf_{G\in \beth_{n}(\Sigma)}\frac{w(G)}{\mathcal{MK}(G,\infty)}\quad \textit{and}\quad c_{6}(w)=\inf_{G\in \beth_{n}(\Sigma)}\frac{w(G)}{\mathcal{MK}(G,O)}. $$

As an application of Theorem 1 and Lemma 3 in Section 2, we give the growth properties of positive superharmonic functions at infinity in a cone.

### Theorem 2

*Let* $w(G)$ ($\not\equiv+\infty$) ($G=(r,\Xi)\in\beth_{n}(\Sigma)$) *be defined by* (11). *Then*

$$w(G)-c_{5}(w)\mathcal{MK}(G,\infty)-c_{6}(w) \mathcal{MK}(G,O)=o\bigl(r^{\iota _{\Sigma}}\bigr) $$

*for any* $G\in\beth_{n}(\Sigma)- \mathbb{EX}(\epsilon;\xi,n-1)$ *as* $r \rightarrow\infty$, *where* $\mathbb{EX}(\epsilon;\xi,n-1)$ *is a subset of* $\beth_{n}(\Sigma)$ *and has a covering* $\{r_{k},R_{k}\}$ *satisfying* (8).

Theorem 2 immediately gives the following corollary.

### Corollary 4

*Let* $w(x)$ ($\not\equiv+\infty$) ($x=(X,x_{n})\in\mathcal{T}_{n}$) *be defined by* (10). *Then* $w(x)-cx_{n}=o(|x|)$ *for any* $x\in\mathcal{T}_{n}- \mathbb{EX}(\epsilon;\varrho,n-1)$ *as* $|x| \rightarrow\infty$, *where* $\mathbb{EX}(\epsilon;\varrho,n-1)$ *is a subset of* $\beth_{n}(\Sigma )$ *and has a covering satisfying* (8).

## Lemmas

In order to prove our main results we need following lemmas. In this paper let *M* denote various constants independent of the variables in questions, which may be different from line to line.

### Lemma 1

see [4], Lemma 2

*Let any* $G=(r,\Xi)\in\beth_{n}(\Sigma)$ *and any* $H=(t,\Omega)\in \daleth_{n}(\Sigma)$, *we have the following estimates*:

12 $$ \mathcal{POI}_{\Sigma}(G,H)\leq M r^{-\kappa _{\Sigma}}t^{\iota_{\Sigma}-1}h_{\Sigma}( \Xi)\frac{\partial}{\partial n_{\Omega}}h_{\Sigma}(\Omega) $$

*for* $0<\frac{t}{r}\leq\frac{4}{5}$,

13 $$ \mathcal{POI}_{\Sigma}(G,H)\leq M r^{\iota _{\Sigma}}t^{-\kappa_{\Sigma}-1}h_{\Sigma}( \Xi)\frac{\partial }{\partial n_{\Omega}}h_{\Sigma}(\Omega) $$

*for* $0<\frac{r}{t}\leq\frac{4}{5}$, *and*

14 $$ \mathcal{POI}_{\Sigma}(G,H)\leq Mh_{\Sigma}(\Xi )t^{1-n}\frac{\partial}{\partial n_{\Omega}}h_{\Sigma}(\Omega )+Mrh_{\Sigma}( \Xi)|G-H|^{-n}\frac{\partial}{\partial n_{\Omega}}h_{\Sigma}(\Omega) $$

*for* $\frac{4r}{5}< t\leq\frac{5r}{4}$.

### Lemma 2

see [5], Lemma 5

*If* $\beta\geq0$ *and* *λ* *is positive measure on* $\mathbf{R}^{n}$ *having finite total mass*, *then exceptional set* $\mathbb{EX}(\epsilon; \lambda, \beta)$ *has a covering* $\{r_{k},R_{k}\}$ ($k=1,2,\ldots$) *satisfying*

$$\sum_{k=1}^{\infty}\biggl(\frac{r_{k}}{R_{k}} \biggr)^{\beta}< \infty. $$

The estimation of the Green potential at infinity is the following, which is due to [5].

### Lemma 3

*If* *ν* *is a positive measure on* $\beth_{n}(\Sigma)$ *such that* (1) *holds for any* $G\in\beth _{n}(\Sigma)$. *Then*

$$\mathcal{GF}_{\Sigma} \nu(G)=o\bigl(r^{\iota_{\Sigma}}\bigl\{ h_{\Sigma}(\Xi)\bigr\} ^{1-\alpha}\bigr) $$

*for any* $G=(r,\Xi)\in \beth_{n}(\Sigma)-\mathbb{EX}(\epsilon;\nu',n-\alpha)$ *as* $r \rightarrow \infty$, *where* $\mathbb{EX}(\epsilon;\nu',n-\alpha)$ *is a subset of* $\beth_{n}(\Sigma)$ *and has a covering* $\{r_{k},R_{k}\}$ *satisfying* (5).

## Proof of Theorem 1

Let $G=(r,\Xi)$ be any point in the set $\beth _{n}(\Sigma; (L,+\infty))-\mathbb{EX}(\epsilon; \mu', n-\alpha)$, where *r* is a sufficiently large number satisfying $r\geq\frac{5l}{4}$.

Put

$$\mathcal{POI}_{\Sigma}\mu(G)=\mathcal{POI}_{\Sigma}^{1}(G)+ \mathcal {POI}_{\Sigma}^{2}(G)+\mathcal{POI}_{\Sigma}^{3}(G), $$

where

$$\begin{aligned} &\mathcal{POI}_{\Sigma}^{1}(G)= \int_{\daleth_{n}(\Sigma;(0,\frac {4}{5}r])}\mathcal{POI}_{\Sigma}(G,H)\,d\mu(H),\\ &\mathcal{POI}_{\Sigma}^{2}(G)= \int_{\daleth_{n}(\Sigma;(\frac {4}{5}r,\frac {5}{4}r))}\mathcal{POI}_{\Sigma}(G,H)\,d\mu(H), \\ &\mathcal{POI}_{\Sigma}^{3}(G)= \int_{\daleth_{n}(\Sigma;[\frac {5}{4}r,\infty ))}\mathcal{POI}_{\Sigma}(G,H)\,d\mu(H). \end{aligned} $$

We have the following estimates:

15 $$\begin{aligned} & \mathcal{POI}_{\Sigma}^{1}(G) \leq Mr^{\iota_{\Sigma}}h_{\Sigma}(\Xi) \biggl(\frac{4}{5}r \biggr)^{-\varrho_{\Sigma}} \int _{\daleth_{n}(\Sigma;(0,\frac{4}{5}r])}t^{\iota_{\Sigma}-1}\frac {\partial }{\partial n_{\Omega}}h_{\Sigma}( \Omega)\,d\mu(H) \\ &\hphantom{\mathcal{POI}_{\Sigma}^{1}(G)}\leq M \epsilon r^{\iota_{\Sigma}}h_{\Sigma}(\Xi), \end{aligned}$$

16 $$\begin{aligned} & \mathcal{POI}_{\Sigma}^{3}(G) \leq Mr^{\iota_{\Sigma}}h_{\Sigma}(\Xi) \int_{\daleth_{n}(\Sigma;[\frac {5}{4}r,\infty))}t^{-\kappa_{\Sigma}-1}\frac{\partial}{\partial n_{\Omega}}h_{\Sigma}( \Omega)\,d\mu(H) \\ &\hphantom{\mathcal{POI}_{\Sigma}^{3}(G)}\leq M \epsilon r^{\iota_{\Sigma}}h_{\Sigma}(\Xi), \end{aligned}$$

from (12), (13), and [11], Lemma 4.

By (14), we write

$$\mathcal{POI}_{\Sigma}^{2}(G)\leq\mathcal{POI}_{\Sigma }^{21}(G)+ \mathcal {POI}_{\Sigma}^{22}(G), $$

where

$$\begin{aligned} &\mathcal{POI}_{\Sigma}^{21}(G)=M \int_{\daleth_{n}(\Sigma;(\frac {4}{5}r,\frac{5}{4}r))}t^{\kappa_{\Sigma}+1}h_{\Sigma}(\Xi)t^{1-n}\,d \mu'(H),\\ &\mathcal{POI}_{\Sigma}^{22}(G)=M \int_{\daleth_{n}(\Sigma;(\frac {4}{5}r,\frac{5}{4}r))}t^{\kappa_{\Sigma}+1}rh_{\Sigma}(\Xi )|G-H|^{-n}\,d\mu'(H). \end{aligned} $$

We first have

17 $$\begin{aligned} \mathcal{POI}_{\Sigma}^{21}(G) \leq& Mr^{\iota_{\Sigma}}h_{\Sigma}(\Xi) \int_{\daleth_{n}(\Sigma;(\frac {4}{5}r,\infty))}d\mu'(H) \\ \leq& M \epsilon r^{\iota_{\Sigma}}h_{\Sigma}(\Xi) \end{aligned}$$

from [11], Lemma 4.

Next, we shall estimate $\mathcal{POI}_{\Sigma}^{22}(G)$. We can find a number $k_{1}$ satisfying $k_{1}\geq0$ and

$$\daleth_{n}\biggl(\Sigma;\biggl(\frac{4}{5}r, \frac{5}{4}r\biggr)\biggr)\subset B\biggl(G,\frac{r}{2}\biggr) $$

for any $G=(r,\Xi)\in\Lambda(k_{1})$, where

$$\Lambda(k_{1})=\Bigl\{ G=(r,\Xi)\in\beth_{n}(\Sigma); \inf _{z\in\partial \Sigma }\bigl|(1,\Xi)-(1,z)\bigr|< k_{1}, 0< r< \infty\Bigr\} . $$

Then the set $\beth_{n}(\Sigma)$ can be split into two sets $\Lambda (k_{1})$ and $\beth_{n}(\Sigma)-\Lambda(k_{1})$.

Let $G=(r,\Xi)\in\beth_{n}(\Sigma)-\Lambda(k_{1})$. Then

$$|G-H|\geq k_{1}'r, $$

where $H\in \daleth_{n}(\Sigma)$ and $k_{1}'$ is a positive number. So

18 $$\begin{aligned} \mathcal{POI}_{\Sigma}^{22}(G) \leq& Mr^{\iota_{\Sigma}}h_{\Sigma}(\Xi) \int_{\daleth_{n}(\Sigma;(\frac {4}{5}r,\infty))}d\mu'(H) \\ \leq& M \epsilon r^{\iota_{\Sigma}}h_{\Sigma}(\Xi) \end{aligned}$$

from [11], Lemma 4.

If $G\in\Lambda(k_{1})$, we put

$$F_{l}(G)=\biggl\{ H\in\daleth_{n}\biggl(\Sigma;\biggl( \frac{4}{5}r,\frac{5}{4}r\biggr)\biggr); 2^{l-1}\varrho(G) \leq|G-H|< 2^{l}\varrho(G)\biggr\} . $$

Since $\daleth_{n}(\Sigma)\cap\{H\in\mathbf{R}^{n}: |G-H|< \varrho(G)\}=\varnothing$, we have

$$\mathcal{POI}_{\Sigma}^{22}(G)=M\sum _{i=1}^{l(G)} \int _{F_{l}(G)}t^{\kappa _{\Sigma}+1}rh_{\Sigma}( \Xi)|G-H|^{-n}\,d\mu'(H), $$

where $l(G)$ is a positive integer satisfying $2^{l(G)-1}\varrho(G)\leq\frac{r}{2}<2^{l(G)}\varrho(G)$.

By Remark 3 we have $rh_{\Sigma}(\Xi)\leq M\varrho(G)$ ($G=(r,\Xi)\in\beth_{n}(\Sigma)$), and hence

$$\begin{aligned} \int_{F_{l}(G)}\frac{t^{\kappa_{\Sigma}+1}rh_{\Sigma}(\Xi )}{|G-H|^{n}}\,d\mu'(H) \leq& Mr^{\kappa_{\Sigma}-\alpha+2}\bigl\{ h_{\Sigma}(\Xi)\bigr\} ^{1-\alpha}\mu '\bigl(F_{l}(G)\bigr)\bigl\{ 2^{l}\varrho(G) \bigr\} ^{\alpha-n} \end{aligned}$$

for $l=0,1,2,\ldots,l(G)$.

Since $G=(r,\Xi)\notin\mathbb{EX}(\epsilon; \mu', n-\alpha)$, we have

$$\mu'\bigl(F_{l}(G)\bigr)\bigl\{ 2^{l}\varrho(G) \bigr\} ^{\alpha-n}\leq\mu'\bigl(B\bigl(G,2^{l}\varrho (G)\bigr)\bigr)\bigl\{ 2^{l}\varrho(G)\bigr\} ^{\alpha-n}\leq \mathfrak{M}\bigl(G; \mu', n-\alpha\bigr)\leq \epsilon r^{\alpha-n} $$

for $l=0,1,2,\ldots,l(G)-1$ and

$$\mu'\bigl(F_{l(G)}(G)\bigr)\bigl\{ 2^{l}\varrho(G) \bigr\} ^{\alpha-n}\leq\mu'\biggl(B\biggl(G,\frac {r}{2} \biggr)\biggr) \biggl(\frac{r}{2}\biggr)^{\alpha-n}\leq\epsilon r^{\alpha-n}. $$

So

19 $$ \mathcal{POI}_{\Sigma}^{22}(G)\leq M \epsilon r^{\iota_{\Sigma}}\bigl\{ h_{\Sigma}(\Xi)\bigr\} ^{1-\alpha}. $$

From (15), (16), (17), (18), (19), and Remark 4, we obtain $\mathcal{POI}_{\Sigma}\mu(G)=o(r^{\iota_{\Sigma}}\{h_{\Sigma}(\Xi)\} ^{1-\alpha})$ for any $G=(r,\Xi)\in\beth_{n}(\Sigma; (L,+\infty))-\mathbb{EX}(\epsilon; \mu', n-\alpha)$ as $r\rightarrow\infty$, where *L* is a sufficiently large real number. With Lemma 3 we have the conclusion of Theorem 1.

## Proof of Corollary 1

Let $G=(r,\Xi) $ be a fixed point in $\beth_{n}(\Sigma)$. Then there exists a number *R* satisfying $\max\{\frac{5r}{4},1\}< R$. There exists a positive constant $M'$ such that

20 $$ \mathcal{POI}_{\Sigma}(G,H)\leq M' r^{\iota_{\Sigma}}t^{-\kappa _{\Sigma}-1}h_{\Sigma}(\Xi) $$

from Remark 2 and (13), where $H=(t,\Omega)\in\daleth _{n}(\Sigma )$ satisfying $0<\frac{r}{t}\leq \frac{4}{5}$.

Let $M=M'c_{n}^{-1}r^{\iota_{\Sigma}}h_{\Sigma}(\Xi)$. Then we have from (6) and (20)

$$\begin{aligned} \int_{\daleth_{n}(\Sigma;(R,+\infty))}\bigl|g(H)\bigr|\mathcal{POI}_{\Sigma }(G,H)\,d \sigma_{H} \leq& M \int_{R}^{\infty}t^{-\iota_{\Sigma}-1}\biggl( \int_{\partial{\Sigma }}\bigl|g(t,\Omega )\bigr|\,d_{\sigma_{\Omega}}\biggr)\,dt< \infty. \end{aligned}$$

For any $G\in\beth_{n}(\Sigma)$, it is easy to see that $\mathcal {POI}_{\Sigma}[g](G)$ is finite, which means that $\mathcal{POI}_{\Sigma}[g](G)$ is a harmonic function of $G\in \beth_{n}(\Sigma)$. Meanwhile, Theorem 1 gives (7). The proof of Corollary 1 is completed.
